# Perioperative Treatment of Metachronous Multiple Lung Cancer with Organizing Pneumonia: A Case Report

**DOI:** 10.5761/atcs.cr.25-00089

**Published:** 2025-08-14

**Authors:** Hiroshi Takehara, Ken Kodama, Toru Momozane, Kansuke Kido

**Affiliations:** 1 Department of Thoracic Surgery, Yao Municipal Hospital, Yao, Osaka, Japan; 2 Department of Pathology, Osaka University Graduate School of Medicine, Suita, Osaka, Japan

**Keywords:** organizing pneumonia (OP), metachronous multiple lung cancer, lobectomy, one-lobe ventilation anesthesia, pulse steroid therapy

## Abstract

We report a rare case of the independent coexistence of lung cancer and organizing pneumonia (OP) in different lobes of the right lung in a 67-year-old man with a history of left upper lobectomy. Computed tomography revealed a lesion consistent with OP in the right upper lobe and a suspicious shadow in the right lower lobe, which was diagnosed as squamous cell carcinoma via bronchoscopic biopsy. The patient underwent right lower lobectomy and partial resection of the right upper lobe under left one-lung ventilation. Empirical corticosteroids were administered preoperatively, followed by a brief postoperative course for 3 days after pathological confirmation of OP to reduce complications such as bronchial fistula, then transitioned to macrolide therapy for 3 months. Fourteen months postoperatively, OP had not recurred, although bone metastases developed and responded well to chemoradiotherapy. This case highlights the need for individualized perioperative management in patients with complex pulmonary pathology.

## Abbreviations

CAM
: clarithromycin
CRP
: C-reactive protein
ECOG-PS
: Eastern Cooperative Oncology Group performance status
MB-LL
: left main bronchus and the left lower lobe bronchus
OP
: organizing pneumonia
POD
: postoperative day
T-MB
: trachea and the left main bronchus

## Introduction

Organizing pneumonia (OP), also known as bronchiolitis obliterans organizing pneumonia, is a clinicopathological syndrome characterized by the presence of intraluminal plugs of connective tissue (Masson bodies) in the bronchioles, alveolar ducts, and alveolar spaces. According to a study by Romero et al.,^1)^ OP in the vicinity of lung neoplasms was found in 33 out of 89 patients (37%). Male sex, smoking, epidermoid histological type, and the presence of lipid pneumonia were found to be significantly more common in patients with OP. These cases associated with a specific etiology are often referred to as secondary OP, which should be differentiated from cryptogenic OP, where no identifiable cause is found.^2)^

We encountered a patient who developed both a second primary lung cancer and OP in separate lobes of the right lung, with the OP not adjacent to the tumor, following the discontinuation of postoperative follow-up after undergoing left upper lobectomy for the initial lung cancer. In this report, we describe the perioperative management strategies for this rare combination of conditions.

## Case Presentation

A 67-year-old man was admitted to our hospital with progressive dyspnea on exertion and recurrent fever over the past 10 days. The patient had a history of left upper lobectomy for pathological-T1cN0M0, stage IA3 (8th edition) lung squamous cell carcinoma 10 years ago in our department. He had been a heavy smoker for 82 pack-years but had subsequently quit smoking. Regular postoperative follow-up was completed 5 years later, and no abnormal shadows were observed on chest radiographs at that time. He had no comorbidities such as diabetes mellitus, autoimmune disease, or chest trauma.

Upon presentation, the patient’s respiratory rate was 20 breaths per minute, and his oxygen saturation was 91% on ambient air. He was febrile (38.0°C), with a heart rate of 97 beats per minute and an arterial blood pressure of 128/75 mmHg. His Eastern Cooperative Oncology Group performance status (ECOG-PS) was 3.

Blood tests revealed elevated serum lactate dehydrogenase of 332 IU/L (124–222), C-reactive protein (CRP) of 11.46 mg/dL (<0.14), KL-6 of 727 IU/mL (<500), neuron-specific enolase of 17.3 ng/mL (<16.3), cytokeratin 19 fragment (CYFRA) of 6.9 ng/mL (<3.5), and decreased hemoglobin of 11.8 g/dL (13.5–17.6). Blood and sputum cultures showed no growth after 72 hours. Coronavirus disease 2019 polymerase chain reaction was negative. Serum β-d-glucan, *Aspergillus galactomannan*, *Capilia Mycobacterium avium* complex antibody enzyme-linked immunosorbent assay, and T-SPOT (Oxford Immunotec, Kanagawa, Japan). TB results were also negative.

**Fig. 1:**
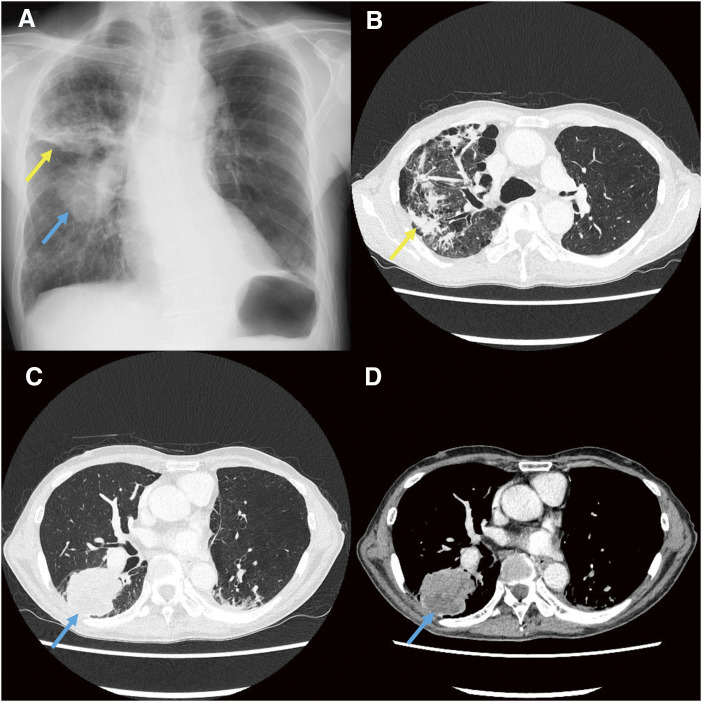
Chest radiograph and chest CT on admission. (**A**) Radiograph showing the independent existence of an infiltrate (yellow arrow) and a noticeable round mass (blue arrow) on the right hemithorax. (**B**) Contrast-enhanced CT (lung window setting) showing a patchy and irregular peripheral and peribronchial consolidation (yellow arrow) at the level of the upper lobes. (**C**) CT showing a 6-cm round tumor (blue arrow) at the level of the lower lobe. (**D**) CT (mediastinal window setting) showing a large low-density area (blue arrow) in the tumor. CT: computed tomography

Chest radiograph (**Fig. 1A**) and chest CT (**Fig. 1B**) at admission showed patchy and linear peribronchial shadows in the right upper lobe and a mass lesion measuring approximately 6 cm in its greatest dimension in the superior segment (S6) of the right lower lobe (**Figs. 1A**–**1D**). There was no apparent mediastinal or hilar lymphadenopathy (clinically T3N0M0, stage IIB).

The patient underwent bronchoscopy, which revealed narrowing of the orifice of the superior segmental bronchus (B6) in the right lower lobe. The tissue obtained by transbronchial biopsy showed squamous cell carcinoma. Cultures of bronchoalveolar lavage specimens from the posterior segment (S2) of the right upper lobe were negative, with no significant cell patterns observed.

Based on the diagnosis of squamous cell carcinoma of the right lower lobe and suspected OP in the right upper lobe, we initiated pulse corticosteroid therapy, administering intravenous methylprednisolone at 1000 mg/day for 3 days, followed by prednisolone at 60 mg/day for an additional 4 days, alongside intravenous levofloxacin at 500 mg/day as the first step of treatment.

Although the CRP level improved and inflammation was considered to have subsided, the pneumonia shadow remained almost unchanged on imaging. However, due to concerns regarding potential cancer progression, we proceeded with radical resection as the second step of treatment, performing a right lower lobectomy 18 days after admission. The preoperative pulmonary function test showed a moderate restrictive pattern as follows (percentage predicted value in parentheses): forced vital capacity (FVC), 2.49 L (65.2%); forced expiratory volume in 1 second (FEV1), 1.98 L (63.3%); and FEV1/FVC, 79.52%.

**Fig. 2:**
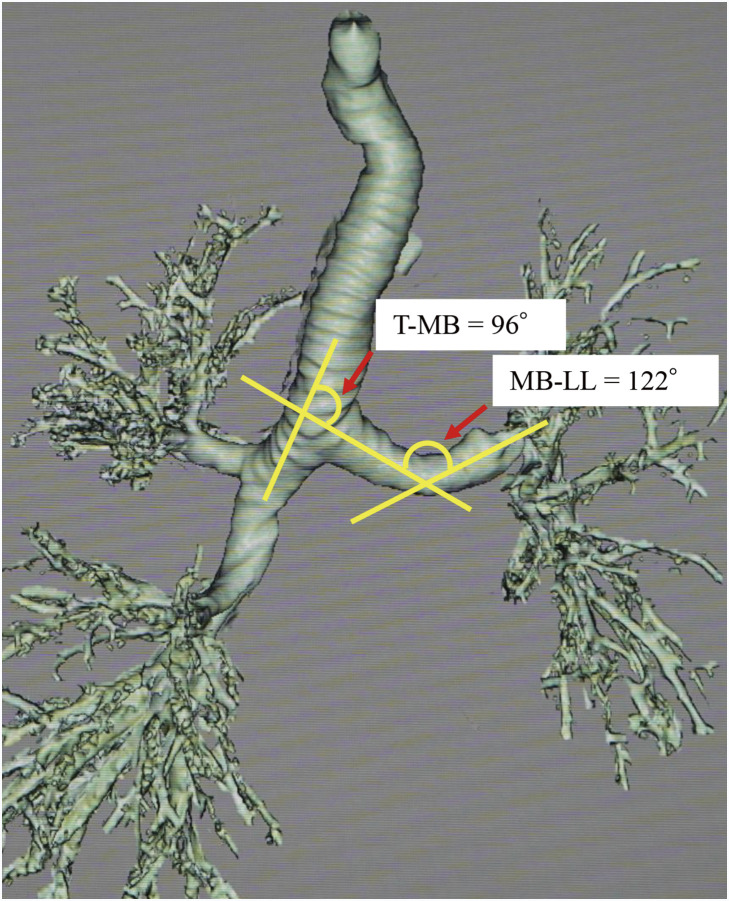
In the present case, 2 angles between the T-MB angle and between the MB-LL angle measured prior to right lung surgery were 96° and 122°, respectively. For these values of angulation, one-lung ventilation anesthesia of the left lower lobe is considered acceptable with a left-sided double-lumen tube.^3)^ MB-LL: left main bronchus and the left lower lobe bronchus; T-MB: trachea and the left main bronchus

**Fig. 3:**
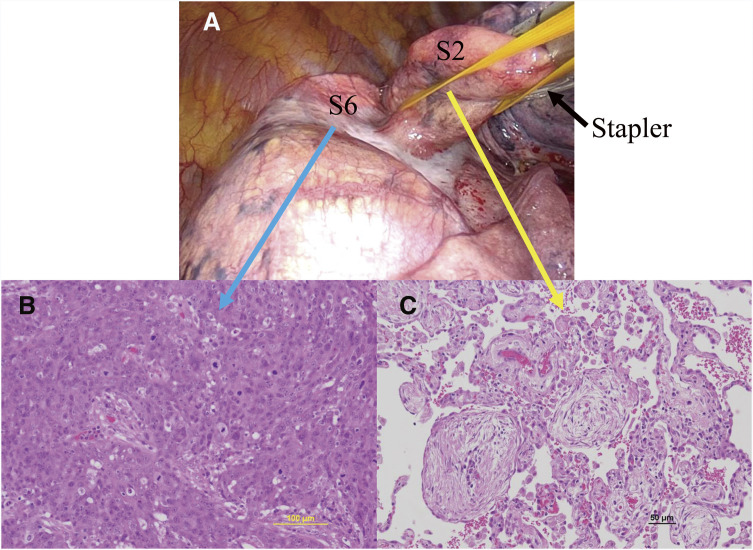
(**A**) Intraoperative photograph showing the partial resection of S2 tightly adhered to S6 using a stapler. (**B**) Photomicrograph of the S6 tumor (H–E stain) showing poorly differentiated squamous cell carcinoma, and keratinization and intercellular bridges were noted in focal areas (inset). (**C**) Photomicrograph of the S2 lesion (H–E stain) showing alveoli filled with loose tissue forming a polypoid formation (Masson body) extending into the respiratory tracts with a small diffuse inflammatory infiltrate in the interalveolar space. H–E: hematoxylin and eosin

The absence of marked bronchial angulation^3)^ due to the previous left upper lobectomy was confirmed on chest CT (**Fig. 2**), allowing us to achieve one-lobe (rather than one-lung) ventilation anesthesia with a left-sided double-lumen endotracheal tube. Through an anterolateral thoracotomy at the 4th intercostal space, we performed a right lower lobectomy and partial resection of the posterior segment (S2) in the upper lobe, which was tightly adhered to the superior segment (S6) of the lower lobe, with systematic lymph node dissection (**Fig. 3A**). Gross pathology revealed a grayish-white tumor with irregular margins in the right lower lobe. Histologically, atypical cells were arranged in a solid pavement-like pattern, with keratinization and intercellular bridges observed in focal areas. Abundant mitotic figures were noted (**Fig. 3B**), and necrosis spread in a map-like pattern. Lipoid pneumonia was found to extend beyond the tumor. Pathologically, the tumor was diagnosed as T3N0M0 stage IIB squamous cell carcinoma. Meanwhile, pathologic examination of the partially resected upper lobe tissue revealed buds of granulation tissue consisting of fibroblasts and myofibroblasts embedded in connective tissue (Masson bodies) (**Fig. 3C**). The granulation tissue extended from one alveolus to another through the interalveolar pores, consistent with OP.

**Fig. 4:**
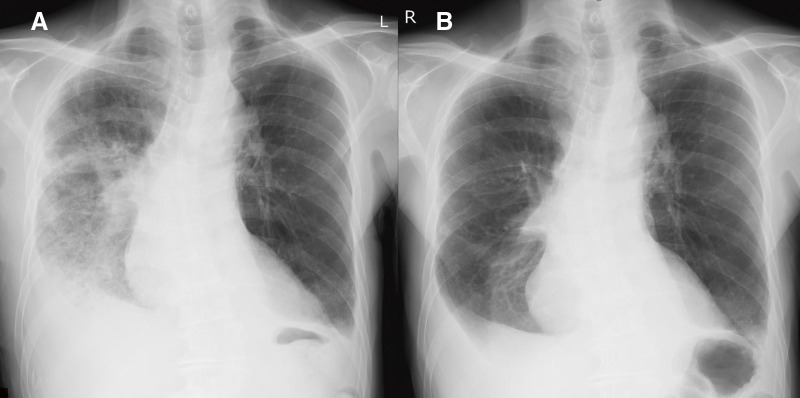
Postoperative radiograph. (**A**) Chest radiography taken on POD 6, before pulse steroid therapy, showing the infiltrate extending into the right residual lung. (**B**) Chest radiography taken on POD 77 showing recovery of the right lung from pneumonia. POD: postoperative day

Based on the pathological diagnosis of OP, postoperative empirical treatment with meropenem (1 g intravenously 3 times a day for 7 days) was initiated. As the final step of treatment, mini-pulse corticosteroid therapy was started on postoperative day 7 (POD 7), with intravenous methylprednisolone at 500 mg/day for 3 days to control the OP extending to the right upper lobe (**Fig. 4A**). Oral clarithromycin (CAM) at 200 mg twice daily was also started on POD 7 and continued for 3 months. The patient was discharged on POD 20, and his OP had completely resolved 4 months postoperatively (**Fig. 4B**).

Six months after surgery, the patient underwent radiation therapy (30 Gy in 10 fractions) for left pelvic and cervical vertebral metastases, followed by 4 cycles of carboplatin and paclitaxel chemotherapy and pembrolizumab-based immunotherapy. He then received 6 cycles of maintenance therapy with pembrolizumab alone and is currently symptom-free with ECOG-PS of 1. The recent pulmonary function test showed the following (percentage predicted value in parentheses): FVC, 2.53 L (66.9%); FEV1, 2.06 L (66.7%); and FEV1/FVC, 81.42%.

## Discussion

This case involved a second lung cancer diagnosed 9 years and 6 months after the first lung cancer and 4 years and 10 months after discontinuing follow-up. The second cancer was an aggressive squamous cell carcinoma characterized by abundant mitotic figures, poor differentiation, and marked necrosis compared to the first cancer. Considering the length of the interval and histologic findings, it is compatible with metachronous primary lung cancer rather than late metastasis.^4)^ The second lung cancer was incidentally discovered when the patient visited our hospital with severe respiratory symptoms due to pneumonia. The diagnosis of OP was made histologically with a piece of tissue from the right upper lobe pneumonia area that was removed during lung cancer surgery. Transbronchial lung biopsy specimens may be insufficient for diagnosing OP; a sufficient specimen obtained by open lung biopsy remains the gold standard.^1,5)^ We were able to make the diagnosis of OP using the specimen obtained from the partially resected right upper lobe. Given the patient’s male sex, smoking history, and the presence of lipid pneumonia around the lung cancer in the right lower lobe, it is reasonable to consider the OP in the right upper lobe as secondary OP, rather than cryptogenic OP.^1,2,6)^

The second cancer appeared as a large solid mass on CT with a large low-density area reminiscent of necrosis. Tumor necrosis, a sign of aggressive tumor growth, has been associated with poorer prognosis in N0 disease.^7)^ Given the rapid progression of the lung cancer, surgery was performed after CRP levels improved, although the pneumonia image did not improve on CT. To secure the surgical field and prevent right lung secretions from sagging, one-lobe ventilation, rather than one-lung ventilation anesthesia, was required. Fortunately, SpO_2_ was maintained at a minimum of 88% during one-lobe ventilation, and the surgery was successfully completed. Kawagoe et al.^3)^ reported that in 18 patients who underwent right lung surgery after a left upper lobectomy, the minimum SpO_2_ during left one-lobe ventilation was 90.9 ± 4.1%. In 2 of 18 patients, intermittent bilateral ventilation was required to prevent desaturation. They concluded that the presence or absence of significant bronchial angulation—defined as a combination of a wide angle (>140°) between the trachea and the left main bronchus and a narrow angle (<100°) between the left main bronchus and the left lower lobe bronchus—critically influenced the choice of airway device, whether a left-sided double-lumen tube, a right-sided double-lumen tube, or a bronchial blocker. In the present case, the 2 measured angles prior to surgery were 96° and 122°, respectively (**Fig. 2**).

In this case, a pulmonary ventilation–perfusion) scan to assess split lung function was not performed. However, preoperative contrast-enhanced CT showed no evidence of pulmonary artery embolism or stenosis, and no active pneumonia in the left lower lobe. These findings suggest that the function of the left lower lobe—the dependent lung during one-lung ventilation—was likely preserved, despite the volume discrepancy between the left and right lungs. Compared to the preoperative pulmonary function, a slight improvement was observed in the most recent pulmonary function test following right lower lobectomy. This is presumed to be the result of improved pulmonary function due to the treatment of OP in the right upper lobe, which outweighed the functional loss caused by the right lower lobectomy (**Fig. 4**).

Although most patients with OP respond to corticosteroid therapy, the optimal dosage and duration of treatment have not been established in clinical trials. Relapse is a common yet unpredictable complication, particularly during corticosteroid tapering or following discontinuation.^2)^ In the present case, it was necessary to treat residual OP in the right upper lobe after the right lower lobectomy. However, the use of corticosteroids in the perioperative period following pulmonary resection may impair wound healing and, in severe cases, result in bronchial fistula formation.^8)^ Therefore, we administered intravenous methylprednisolone at a dose of 500 mg daily for 3 consecutive days without tapering. Instead, based on previous reports suggesting the anti-inflammatory properties of macrolides,^9)^ we prescribed CAM at 200 mg twice daily for 3 months. The patient’s postoperative course was uneventful, and no recurrence of OP has been observed to date.

## Conclusion

We encountered a patient with a second primary lung cancer and OP coexisting in the right lung 10 years after left upper lobectomy for the first primary lung cancer. The patient was successfully treated with right lower lobectomy combined with partial resection of the right upper lobe under one-lobe ventilation anesthesia, followed by minimized postoperative steroid administration based on an accurate pathological diagnosis of OP.

## Declarations

### Funding

None of the authors have any financial disclosures.

### Authors’ contributions

Hiroshi Takehara: Conceptualization; investigation; resources; validation; writing—original draft; writing—review and editing. Ken Kodama: Conceptualization; investigation; resources; supervision; writing—original draft; writing—review and editing. Toru Momozane: Investigation; resources; writing—review and editing. Kansuke Kido: Investigation; resources; writing—review and editing. All authors have read and approved the final version of the manuscript.

### Date availability

Data that support the findings of this study are available from the corresponding author upon reasonable request.

### Ethics approval

This report was approved by the ethics committee of Yao Municipal Hospital (approval no. 100624-213).

### Consent for publication

Written informed consent was obtained from the patient.

### Disclosure statement

All authors have declared no conflict of interest.
